# Spread of ST348 *Klebsiella pneumoniae* Producing NDM-1 in a Peruvian Hospital

**DOI:** 10.3390/microorganisms8091392

**Published:** 2020-09-11

**Authors:** Maria J. Pons, Marta Marí-Almirall, Barbara Ymaña, Jeel Moya-Salazar, Laura Muñoz, Sharon Sauñe, Richard Salazar-Hernández, Jordi Vila, Ignasi Roca

**Affiliations:** 1 Facultad de Medicina, Universidad Científica del Sur, Carr. Antigua Panamericana Sur 19, Villa El Salvador, Lima 15067, Peru; barbara.ymana@gmail.com; 2 Department of Clinical Microbiology, ISGlobal, Hospital Clínic, Universitat de Barcelona, Rosselló 149-153, 08036 Barcelona, Spain; marta.mari@isglobal.org (M.M.-A.); laura.munoz@isglobal.org (L.M.); jvila@ub.edu (J.V.); 3 Hospital Nacional Docente Madre Niño San Bartolomé, Avenida Alfonso Ugarte 825, Lima 150101, Peru; jeel.moya@upsjb.edu.pe; 4 Escuela de Posgrado, Facultad de Ciencias de la Salud, Universidad Privada San Juan Bautista, Ex Hacienda Villa, Av José Antonio Lavalle s/n, Lima 15067, Peru; sharon.saune@upsjb.edu.pe; 5 Servicio de Citología y Citogenética, Departamento de Anatomía Patológica, Hospital Nacional Guillermo Almenara Irigoyen, Jirón García Naranjo 840, La Victoria 13, Lima 150115, Peru; richard.sh30@gmail.com

**Keywords:** carbapenem resistance, metallo-β-lactamase, *Klebsiella pneumoniae*, epidemiology, bloodstream infections

## Abstract

The aim of this study was to characterize carbapenem-resistant *Klebsiella pneumoniae* (CR-Kp) isolates recovered from adults and children with severe bacteremia in a Peruvian Hospital in June 2018. Antimicrobial susceptibility was determined by disc/gradient diffusion and broth microdilution when necessary. Antibiotic resistance mechanisms were evaluated by PCR and DNA sequencing. Clonal relatedness was assessed using pulsed-field gel electrophoresis (PFGE) and multilocus sequence typing (MLST). Plasmid typing was performed with a PCR-based method. Thirty CR-Kp isolates were recovered in June 2018. All isolates were non-susceptible to all β-lactams, ciprofloxacin, gentamicin and trimethoprim-sulfamethoxazole, while mostly remaining susceptible to colistin, tigecycline, levofloxacin and amikacin. All isolates carried the *bla*_NDM-1_ gene and were extended spectrum β-lactamase (ESBL) producers. PFGE showed four different pulsotypes although all isolates but two belonged to the ST348 sequence type, previously reported in Portugal. *bla*_NDM-1_ was located in an IncFIB-M conjugative plasmid. To our knowledge, this is the first report of an New Delhi metallo-β-lactamase (NDM)-producing *K. pneumoniae* recovered from both children and adults in Lima, Peru, as well as the first time that the outbreak strain ST348 is reported in Peru and is associated with NDM. Studies providing epidemiological and molecular data on CR-Kp in Peru are essential to monitor their dissemination and prevent further spread.

## 1. Introduction

In recent years, carbapenem-resistant *Klebsiella pneumoniae* (CR-Kp) has emerged as one of the major multidrug-resistant bacterial pathogens worldwide and has been classified as an urgent threat to public health [1,2]. Until recently, resistance to carbapenems in *Klebsiella* spp. from nosocomial sources was usually mediated by the expression of carbapenem-hydrolyzing *Klebsiella pneumoniae* Carbapenemase (KPC) [3]. During the last decade, however, infections and nosocomial carriers with New Delhi metallo-β-lactamase (NDM)-producing *K. pneumoniae* seem to be emerging [4,5] and have been associated with important clinical outbreaks worldwide [6,7,8], including some countries in Latin America [9]. In this region, the first NDM-producing *K. pneumoniae* isolates were reported from pediatric patients in Guatemala and Colombia in 2011 and 2016, respectively [10,11], and in Peru the first ever NDM-producing Gram negative bacteria reported was *K. pneumoniae* in 2016 [12].

Overall there are limited data on antimicrobial resistance in Peru although the studies available report carbapenem resistance rates greater that 95% in *Acinetobacter baumannii* [13] while *Klebsiella* spp. seem to lead third-generation cephalosporin resistance in the region with resistance rates of up to 75% [14,15]. In this particular scenario, the aim of this study was to analyze the epidemiology and molecular characterization of CR-Kp isolates recovered at a tertiary hospital in Lima, Peru.

## 2. Materials and Methods

### 2.1. Bacterial Samples and Study Population

This study included 30 consecutive clinical isolates of CR-Kp recovered from blood cultures collected from different inpatients with severe sepsis infection at the Guillermo Almenara Irigoyen Hospital in Lima during June 2018. Only the first isolate from each patient was included in the study. Gender, age, ward of isolation and clinical outcomes were recorded for all patients (Table S1).

Blood culture bottles were incubated in an automated system (BACTEC1 9050; Becton Dickinson, Heidelberg, Germany). After incubation, positive blood cultures were subcultured on solid media after Gram staining as appropriate. Pathogens were identified according to conventional microbiology protocols at the Microbiology Laboratory of Guillermo Almenara Irigoyen Hospital, and all *K. pneumoniae* isolates were confirmed by matrix-assisted laser desorption/ionization time-of-flight mass spectrometry (MALDI-TOF/MS) using a Microflex LT benchtop instrument in linear positive mode (Bruker Daltonics, Bremen, Germany).

### 2.2. Antimicrobial Susceptibility Testing

Antimicrobial susceptibility testing was performed by disc diffusion (Becton Dickinson, Heidelberg, Germany) on Mueller–Hinton agar plates in accordance with the Clinical and Laboratory Standards Institute (CLSI) guidelines for the following antimicrobials: meropenem, imipenem, piperacillin-tazobactam, aztreonam, cefotaxime, ceftazidime, amoxicillin-clavulanic acid, trimethoprim-sulfamethoxazole, levofloxacin, ciprofloxacin, gentamicin, amikacin and tigecycline. Susceptibility to colistin was initially screened for using the agar spot test and positives were then assessed by broth microdilution as recommended by the joint CLSI-European Committee on Antimicrobial Susceptibility Testing (EUCAST) polymyxin breakpoints working group [16,17]. The minimum inhibitory concentration (MIC) values for selected strains were also determined by gradient diffusion (Etest, bioMérieux, Solna, Sweden), for the following antibiotics: imipenem, meropenem, ceftazidime, ceftazidime-avibactam, cefotaxime, cefepime, aztreonam, gentamicin, amikacin, tobramycin, ciprofloxacin, levofloxacin, and tigecycline. The MICs and inhibition diameters were interpreted according to CLSI clinical breakpoints and expert rules for *Enterobacterales* (M100-S29) [18], except for tigecycline, which was interpreted using the European Committee on Antimicrobial Susceptibility Testing (EUCAST) breakpoints and rules for *Enterobacterales* (Version 10.0, January 2020) [19]. *Escherichia coli* strain ATCC 25922 was used as a control.

Isolates were categorized as multidrug-resistant (MDR), extensively drug-resistant (XDR), or pandrug-resistant (PDR) according to the following ad hoc definitions; MDR, non-susceptible to at least one antimicrobial agent in three or more antimicrobial categories; XDR, non-susceptible to at least one antimicrobial agent in all but two or fewer antimicrobial categories; PDR, resistant to all the antimicrobial agents tested [20].

### 2.3. Detection and Characterization of Carbapenem Resistance Genes

The phenotypic production of carbapenemases was assessed with the modified Hodge test as well as with meropenem-ethylenediaminetetraacetic acid (EDTA) inhibition assays. At a molecular level, the presence of the following extended spectrum β-lactamase- (ESBL) and carbapenemase-encoding genes was screened by PCR: *bla*_CTX-M_-group 1, *bla*_CTX-M_-group 9, *bla*_KPC_, *bla*_OXA-48_, *bla*_VIM_, *bla*_IMP_, and *bla*_NDM_ genes [21,22]. Amplification products were purified from agarose gels (Spin-PrepTM Gel DNA Kit, San Diego, CA, USA) and sent for Sanger sequencing (Macrogen, Seoul, Korea) whenever necessary. The genetic identity of the *bla*_NDM_ genes was determined upon pairwise sequence alignment with reference sequences retrieved from the Bacterial Beta-Lactamase Alleles Database (https://www.ncbi.nlm.nih.gov/bioproject/305729). The presence of insertion sequences (IS) and other genes flanking *bla*_NDM_ was studied by PCR with specific primers to already known sequences (Table S2). IS structures were identified against the ISfinder database [23].

### 2.4. Transferability and Epidemiological Studies

Conjugation studies were performed by biparental mating assays using the *E. coli* MC1061AziR strain as recipient. Briefly, overnight cultures of donor strains were diluted (1/10) into fresh and pre-warmed Luria–Bertani (LB) broth. Cultures were allowed to grow at 37 °C without shaking to mid-exponential phase (5 × 10^8^ CFU/mL), gently washed in LB medium at room temperature and then mixed with an overnight culture of the recipient strain in a 1:1 ratio. After 4 h of incubation at 37 °C without shaking, transconjugant strains were selected on LB agar plates containing 0.5 mg/L meropenem and 100 mg/L sodium azide and were screened for *bla*_NDM_ by PCR. The incompatibility groups for molecular typing of plasmids were determined using a PCR-based replicon typing kit (PBRT 2.0 kit, Diatheva, Fano, Italy).

The clonal relatedness of bacterial isolates was assessed by pulsed-field gel electrophoresis (PFGE) as described previously [24], using XbaI-digested genomic DNA and a CHEF-DRIII system (Bio-Rad Laboratories, Madrid, Spain). Band profiles were compared using the InfoQuest^TM^ FPv.5.4 software (Bio-Rad Laboratories) and the unweighted pair group method with arithmetic mean to create dendrograms based on the Dice’s similarity coefficient. Bandwidth tolerance and optimization were set at 1.5 and 1%, respectively, and isolates were clustered together if their similarity index was ≥85% [24].

Multilocus sequence typing (MLST) was performed according to the scheme described previously for *K. pneumoniae* [25]. Allele sequences and sequence types (STs) were retrieved from the Institute Pasteur MLST database (https://bigsdb.pasteur.fr/klebsiella/klebsiella.html). The population structure of STs was evaluated using the goeBURST software (http://www.phyloviz.net/goeburst/).

## 3. Results

### 3.1. Ethical Approval

Bacterial samples studied here were recovered from clinical samples used for microbiological diagnosis at clinical microbiology laboratories. Informed consent was, therefore, not required. The protocol for this study was approved by the Ethics Committee on Clinical Research (CEIC) of the Hospital Clinic de Barcelona (HCB/2017/0923, approved 30 November 2017, and HCB/2017/0833, approved 13 November 2017).

### 3.2. Bacterial Samples and Study Population

During June 2018, 30 isolates of CR-Kp were collected from the blood cultures of different inpatients with severe sepsis infection at the Guillermo Almenara Irigoyen Hospital in Lima, Peru. Thirteen patients were male (43.3%, median age of 44), 13 were female (43.3%, median age of 34) and 4 patients were children of less than 12 years of age (13.3%, median age of 8). All of the children recovered successfully, but five adults (three females and two males) died. The majority of the isolates (*n* = 28) were recovered from patients in intensive care units (ICU, children were attended at the corresponding pediatric ICUs) and two isolates were from patients in the burns unit.

### 3.3. Antimicrobial Susceptibility and Characterization of Carbapenem Resistance

Antimicrobial susceptibility testing by disc diffusion showed high resistance rates to most of the antimicrobial agents tested. In particular, all isolates (100%) were non-susceptible to carbapenems (imipenem and meropemen) as well as third-generation cephalosporins, piperacillin-tazobactam, amoxicillin-clavulanic acid, the monobactam aztreonam, trimethoprim-sulfamethoxazole, gentamicin and ciprofloxacin. Susceptibility was only observed to colistin (90%), tigecycline (80%), levofloxacin (76.6%), and amikacin (76.6%) and, hence, all isolates were classified as MDR.

Positive results with the modified Hodge test indicated carbapenemase production, and the presence of metallo-β-lactamases in all strains was confirmed upon EDTA inhibition of carbapenem hydrolysis. PCR screening for β-lactamases yielded negative results for the genes encoding KPC or OXA-48 carbapenemases and was also negative for genes encoding Imipenemase (IMP) or Verona integron-encoded metallo-β-lactamase (VIM) metallo-β-lactamases, but became positive for the presence of the *bla*_NDM_ gene in all isolates. DNA sequencing later confirmed carriage of the *bla*_NDM-1_ allelic variant. Likewise, PCR screening also identified the gene encoding a CTX-M-group 1 β-lactamase in all isolates.

### 3.4. Transferability and Epidemiological Studies

**Figure 1:**
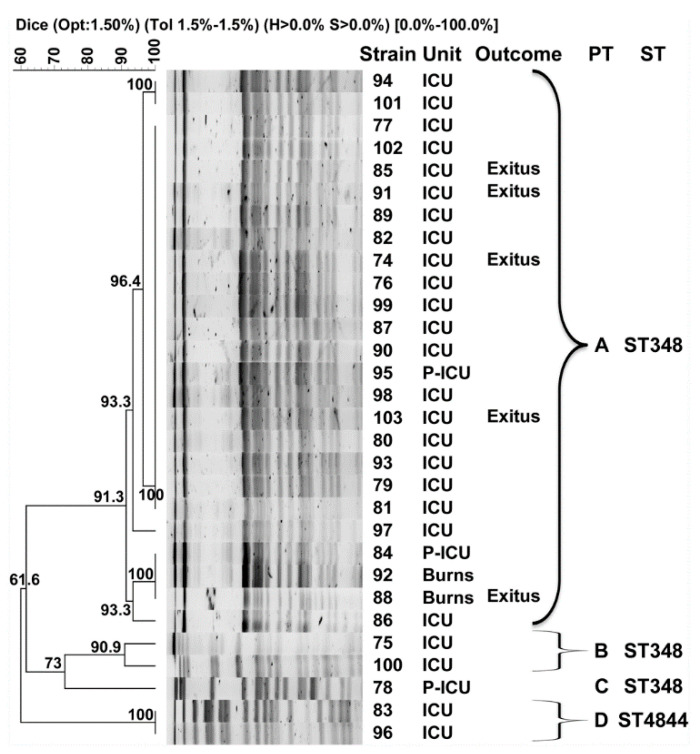
Pulsed-field gel electrophoresis (PFGE) dendrogram of New Delhi metallo-β-lactamase (NDM)-producing *K. pneumoniae* isolates from Guillermo Almenara Irigoyen Hospital in Lima. PT: pulsotype. ST: sequence type. ICU: intensive care unit. P-ICU: pediatric ICU. Braces indicate classification to the corresponding PFGE cluster, or pulsotype. Isolates were included in the same pulsotype if their Dice similarity index was ≥85%.

The analysis of the clonal relatedness of all isolates by PFGE showed the presence of four distinct clusters of strains or pulsotypes, herein designated A to D (Figure 1). Pulsotype A constituted the major cluster with 25 isolates (83.3%) that were grouped together with a similarity index > 91%. The two isolates that were recovered from patients admitted at the burns unit were also included in this cluster. Pulsotypes B and D included two isolates each, also with similarity indexes > 90%, and pulsotype C contained a singleton that was recovered from a pediatric ICU patient. Notably, the CR-Kp isolates recovered from the five deceased patients were all included within the major pulsotype (A), four from the ICU and one from the burns unit. One isolate from each pulsotype was selected for MLST analysis following the Pasteur scheme for *K. pneumoniae* (Table 1). Despite only sharing 61% similarity in the PFGE analysis, isolates from pulsotypes A, B and C belonged to the same sequence type, ST348, while the isolate from pulsotype D presented a new *phoE* allele and was assigned a new ST by the Pasteur curators, that is, ST4844.

The four selected strains were used in transferability studies by biparental mating using a sodium azide resistant *E. coli* strain as the recipient. Transconjugants growing on 0.5 μg/mL meropenem and 100 μg/mL sodium azide were obtained for all four representative strains at estimated transfer frequencies ranging from 6 × 10^−8^ to 3 × 10^−7^ transconjugants per donor. MIC testing showed that all the transconjugants had acquired non-susceptibility to all the β-lactams (including aztreonam) as well as resistance to gentamicin and tobramycin but remained susceptible to all other antimicrobials tested. All of them had also acquired both the *bla*_NDM-1_ and the *bla*_CTX-M_ group 1 genes (Table 1 and Figure S1).

Plasmid typing studies identified IncA/C and several IncF-related plasmid replicons within the four representative strains, but only the IncFIB-M replicon in the transconjugant strains (Table 1), thus suggesting that both *bla*_NDM-1_ and *bla*_CTX-M_ were located in the same conjugative plasmid. In addition, PCR analysis and sequencing of the genetic surrounding of *bla*_NDM-1_ showed the presence of a truncated insertion sequence IS*Aba125* upstream of *bla*_NDM-1_ as well as the highly conserved downstream region that includes the bleomycin resistance protein (BRP_MBL_) gene *ble*, the phosphoribosylanthranilate isomerase protein gene *trpF*, the twin-arginine translocation pathway signal sequence domain protein gene *dsbC*, the periplasmic divalent cation tolerance protein gene *cutA1*, and the truncated chaperonin subunit gene *groES* [4].

## 4. Discussion

NDM was first described in Peru in 2016 from nine *K. pneumoniae* isolates recovered in a single institution [12]. Later in 2018, a multicenter nation-wide study reported 55 NDM-producing *Enterobacterales* recovered between 2013–2017 from different hospitals, which highlighted carriage of *bla*_NDM_ as the predominant carbapenem-resistant mechanism in this country, mostly associated with *K. pneumoniae* [26]. The latter study also showed a steady increase in the number of NDM-producing isolates over time. Nevertheless, data regarding the clonal relatedness or molecular characterization of the isolates were missing in both reports. Molecular data on NDM-producing bacteria were indeed provided in 2018 by Tamariz et al., and in 2019 by Rocha et al., but those studies focused on *E. coli* and *A. baumannii*, respectively [27,28]. Here we report the spread of NDM-producing *K. pneumoniae* causing severe sepsis in ICU patients at a tertiary hospital in Lima, Peru. We identified a major clonal lineage belonging to ST348 that was responsible for 83.3% of the infections and affected male and female patients as well as children. Sporadic isolates belonging to the same sequence type but showing a different PFGE type as well as two isolates from a new ST (ST4844) were also recovered. ST348 is not related to any of the worldwide epidemic clones of *K. pneumoniae*, such as ST258, ST15, ST101 or ST147 [29], but it has been associated with CTX-M-15-producing outbreak strains emerging in Italy and circulating in Portugal since at least 2012 that subsequently acquired *bla*_KPC-3_ [30,31,32]. More recently, a single ST348 isolate producing KPC-5 has also been identified in Quito, Ecuador [33]. The newly identified ST4844 was also not related to any known ST or major clonal groups, according to goeBurst analysis (data not shown).

Interestingly, and compared to other series of patients with NDM-producing *K. pneumoniae* [34,35,36,37], more than half of the adult patients were under 40 years of age and none were older than 55 years old, they were evenly divided between males and females, and four children were also affected. Notably, none of the isolates showed hypermucoviscosity upon performing the string test (data not shown), a trait that is often characteristic (albeit not specific) of hypervirulent strains [38].

In the present study, all of the isolates carried the gene encoding for NDM-1 in a conjugative IncFIB-M plasmid, thus suggesting that while monoclonal spread was the predominant mechanism of dissemination, horizontal gene transfer was also present and allowed the acquisition of *bla*_NDM-1_ by non-clonal strains.

Unfortunately, surveillance data prior to or after the study period as well as data regarding CR-Kp isolates from other sources (e.g., fecal carriers, urinary tract infections or environmental samples) were not available and we could not obtain clinical data related to comorbidities, severity scores or antibiotic treatment either, which we acknowledge as a limitation in the present study.

In conclusion, the results presented here provide a snapshot regarding epidemiological and molecular data on NDM-producing *K. pneumoniae* in Peru. Nevertheless, and given the number and clonality of CR-Kp isolates recovered in just one month from infected patients, this may just be the tip of the iceberg and NDM-producing *K. pneumoniae* are likely to be widespread in this setting, probably associated with the dissemination of ST348 but also with the horizontal transfer of *bla*_NDM-1_ within a conjugative plasmid. Additional studies to evaluate the prevalence of CR-Kp among fecal carriers have been encouraged. To our knowledge, this is the first time that ST348 has been reported in Peru and also the first time that it has been associated with NDM metallo-β-lactamases.

## Figures and Tables

**Table 1:** Antimicrobial susceptibility and molecular characterization of selected *K. pneumoniae* isolates and their corresponding transconjugant *E. coli* strains (*E. coli* MC1061AziR strain was used as recipient).

Strain	MIC (mg/L)														PT	ST	NDM	CTX-M	Inc
	IPM	MEM	CAZ	CZA	CTX	FEP	ATM	GEN	AMK	TOB	CIP	LVX	TGC	CST ^a^					
79	8	16	>256	>256	>32	16	96	32	3	16	2	0.5	2	2	A	348	+	+	A/C, FIIk, FIB-KN, FIB-M
100	12	16	>256	>256	>32	16	16	64	3	32	>32	12	1	2	B	348	+	+	A/C, FIA, FIB, FIB-M
78	16	16	>256	>256	>32	24	24	64	3	16	4	1	1	2	C	348	+	+	A/C, FIIk, FIB-KN, FIB-M
83	4	8	>256	>256	>32	16	32	48	2	16	6	2	4	2	D	4844	+	+	A/C, FIIk, FIB-KN, FIB-M
79t	4	8	>256	>256	>32	16	16	48	4	16	1	0.25	0.25	ND	NA	NA	+	+	FIB-M
100t	8	6	>256	>256	>32	12	12	64	6	16	1	0.25	0.25	ND	NA	NA	+	+	FIB-M
78t	4	4	>256	>256	>32	16	8	96	6	16	0.75	0.25	0.25	ND	NA	NA	+	+	FIB-M
83t	8	8	>256	>256	>32	24	8	64	4	24	1	0.25	0.25	ND	NA	NA	+	+	FIB-M
MC1061	0.19	0.012	0.125	ND	0.047	0.023	0.094	0.25	2	0.25	0.008	0.023	0.25	ND	NA	NA	-	-	-

MIC: minimum inhibitory concentration; IPM: imipenem; MEM: meropenem; CAZ: ceftazidime; CZA: ceftazidime-avibactam; CTX: cefotaxime; FEP: cefepime; ATM: aztreonam; GEN: gentamicin; AMK: amikacin; TOB: tobramycin; CIP: ciprofloxacin; LVX: levofloxacin; TGC: tigecycline; CST: colistin; PT: pulsotype; ST: sequence type; NDM: PCR positive for *bla*_NDM-1_; CTX-M: PCR positive for *bla*_CTX-M_-group 1; Inc: PCR positive for plasmid incompatibility group; ^a^ Colistin’s MIC was determined by broth microdilution. ND: not determined; NA: not applicable. Lower-case *t* letter indicates transconjugant strains.

## Supplementary Materials

The following are available online at https://www.mdpi.com/2076-2607/8/9/1392/s1. Figure S1: Gel electrophoresis; Table S1: Antimicrobial susceptibility and epidemiological features of NDM-producing *K. pneumoniae* isolates recovered from different patients with severe sepsis infection at the Guillermo Almenara Irigoyen Hospital in Lima during June 2018; Table S2: Oligonucleotides used to study the genetic surrounding of *bla*_NDM-1_.
