# Expression of Concern: Pons et al. Spread of ST348 *Klebsiella pneumoniae* Producing NDM-1 in a Peruvian Hospital. *Microorganisms* 2020, *8*, 1392

**DOI:** 10.3390/microorganisms13081739

**Published:** 2025-07-25

**Authors:** Microorganisms Editorial Office

**Affiliations:** MDPI, St. Alban-Anlage 66, 4052 Basel, Switzerland; microorganisms@mdpi.com

With this notice, the Microorganisms Editorial Office alerts the readers to concerns related to this article [1]. Following publication, concerns were raised to the Editorial Office regarding the ethics approval information presented in this study. The Editor-in-Chief has decided to issue this expression of concern while an investigation is being conducted. The authors’ institution has been contacted to contribute to this investigation.

Once this process has been completed, the Editorial Office will provide an update on this situation and announce any post-publication modification deemed to be necessary, as per MDPI’s Update to Published Papers policy.
