# Supplementary material for: Spread of ST348 Klebsiella pneumoniae Producing NDM-1 in a Peruvian Hospital
Source: Microorganisms. 2020 Sep 11;8(9):1392. doi: 10.3390/microorganisms8091392 (PMC7563475; doi:10.3390/microorganisms8091392)
Supplement: Supplementary file 1 [file microorganisms-08-01392-s001.zip › microorganisms-903520_FigureS1.docx]

**Supplementary Material**

**FigS1**

**Figure S1**


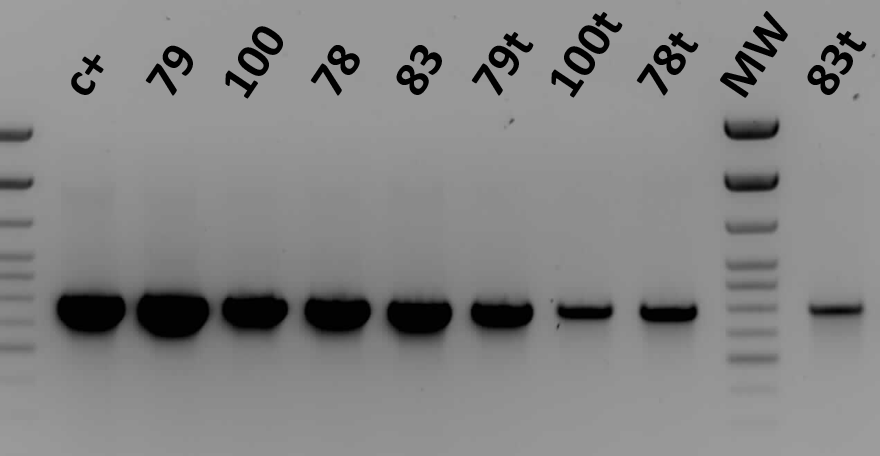


**Figure S1.** Gel electrophoresis (2% agarose, 1xTAE buffer) showing positive PCR amplification bands of roughly 800 bp corresponding to the amplification of *bla*_NDM_ from selected strains and their corresponding transconjugant strains, indicated by lower-case t letters. Primers used were (5’ → 3’): NDM forward: CCA ATA TTA TGC ACC CGG TCG; NDM reverse: ATG CGG GCC GTA TGA GTG ATT G; c+: positive control; MW: 100 bp DNA ladder (top to bottom: 2000 bp, 1500 bp, 1200 bp, 1000 bp, 900 bp, 800 bp, 700 bp, 600 bp, 500 bp, and 400 bp).
