# Supplementary material for: Spread of ST348 Klebsiella pneumoniae Producing NDM-1 in a Peruvian Hospital
Source: Microorganisms. 2020 Sep 11;8(9):1392. doi: 10.3390/microorganisms8091392 (PMC7563475; doi:10.3390/microorganisms8091392)
Supplement: Supplementary file 1 [file microorganisms-08-01392-s001.zip › microorganisms-903520_TableS2.docx]

**Supplementary Material**

**Table S2.** Oligonucleotides used to study the genetic surrounding of *bla*_NDM-1_.

| **Primer** | **Sequence (5’ → 3’)** |
| --- | --- |
| GroESInt-Rv | CAGCTTCTCTTCTTCCATGCGC |
| GroESInt-Fw | GTTCGCCCGGTTCTGTTGTC |
| ISAba125-Fw | GCTTGAGTCAAGAAAAGAAGG |
| ISAba125lw-Fw | GTTGCCATGTCACTGAATAC |
| ISAba125lw2-Fw | CTGTCGCACCTCATGTTTG |
| ISAba125-Rv | CAAACATGAGGTGCGACAG |
| NDMinv2-Fw | TGCCGACACTGAGCACTAC |
| NDMinv2-Rv | GGTCGCCAGTTTCCATTTGC |
| NDM-Fw | CCAATATTATGCACCCGGTCG |
| NDM-Rv | ATGCGGGCCGTATGAGTGATTG |
| Tat2-Fw | ATGACCGCATCCACGATCCG |
| Tat-Rv | CAAAGATCGGCTTGCGGGTG |
| Tat-Fw | GTACAGGTAATAGCCGTCGG |
| TrpF-Fw | GCGGGATCAGCACACCCGAG |
| TrpF2-Rv | CTTGTCGGTATCCTTGACGC |
