# Supplementary material for: Spread of ST348 Klebsiella pneumoniae Producing NDM-1 in a Peruvian Hospital
Source: Microorganisms. 2020 Sep 11;8(9):1392. doi: 10.3390/microorganisms8091392 (PMC7563475; doi:10.3390/microorganisms8091392)
Supplement: Supplementary file 1 [file microorganisms-08-01392-s001.zip › microorganisms-903520_TableS1.docx]

**Supplementary Material**

**Table S1.** Antimicrobial susceptibility and epidemiological features of NDM-producing *K. pneumoniae* isolates recovered from different patients with severe sepsis infection at the Guillermo Almenara Irigoyen Hospital in Lima during June 2018.

| **Strain** | **Gdr** | **Age** | **Ward** | **Outcome** | **PT** | **MLST** | **Antimicrobial susceptibility^a^** | | | | | | | | | | | | | |
| --- | --- | --- | --- | --- | --- | --- | --- | --- | --- | --- | --- | --- | --- | --- | --- | --- | --- | --- | --- | --- |
|  |  |  |  |  |  |  | MEM | IPM | CTX | CAZ | TZP | AMC | ATM | SXT | GEN | CIP | CST | TGC | LVX | AMK |
| 74 | M | 35 | ICU | Exitus | A | 348 | R | R | R | R | R | R | R | R | R | I | S | S | S | I |
| 76 | M | 12 | ICU |  | A | 348 | R | R | R | R | R | R | R | R | R | I | S | S | S | S |
| 77 | F | 45 | ICU |  | A | 348 | R | R | R | R | R | R | R | R | R | I | S | S | S | R |
| 79 | M | 36 | ICU |  | A | 348 | R | R | R | R | R | R | R | R | R | I | S | R | S | S |
| 80 | F | 24 | ICU |  | A | 348 | I | I | R | R | R | R | R | R | R | I | S | S | I | S |
| 81 | M | 30 | ICU |  | A | 348 | R | R | R | R | R | R | R | R | R | I | S | R | S | R |
| 82 | F | 42 | ICU |  | A | 348 | R | R | R | R | R | R | R | R | R | I | S | S | S | S |
| 84 | M | 9 | P-ICU |  | A | 348 | R | R | R | R | R | R | R | R | R | R | S | S | S | S |
| 85 | F | 55 | ICU | Exitus | A | 348 | R | R | R | R | R | R | R | R | R | I | S | S | S | I |
| 86 | M | 47 | ICU |  | A | 348 | R | R | R | R | R | R | R | R | R | I | S | S | S | S |
| 87 | M | 18 | ICU |  | A | 348 | R | R | R | R | R | R | R | R | R | I | S | S | R | S |
| 88 | F | 22 | Burns | Exitus | A | 348 | R | R | R | R | R | R | R | R | R | R | R | S | S | S |
| 89 | M | 39 | ICU |  | A | 348 | R | R | R | R | R | R | R | R | R | R | S | R | S | S |
| 90 | F | 34 | ICU |  | A | 348 | R | R | R | R | R | R | R | R | R | I | S | S | S | S |
| 91 | F | 53 | ICU | Exitus | A | 348 | R | R | R | R | R | R | R | R | R | I | S | S | I | S |
| 92 | M | 44 | Burns |  | A | 348 | R | R | R | R | R | R | R | R | R | I | S | S | S | R |
| 93 | F | 25 | ICU |  | A | 348 | R | R | R | R | R | R | R | R | R | I | S | R | S | S |
| 94 | F | 29 | ICU |  | A | 348 | R | R | R | R | R | R | R | R | R | R | R | S | S | S |
| 95 | F | 5 | P-ICU |  | A | 348 | R | R | R | R | R | R | R | R | R | R | S | R | S | S |
| 97 | M | 47 | ICU |  | A | 348 | R | R | R | R | R | R | R | R | R | I | S | S | S | R |
| 98 | F | 32 | ICU |  | A | 348 | R | R | R | R | R | R | R | R | R | I | S | S | S | R |
| 99 | F | 38 | ICU |  | A | 348 | R | R | R | R | R | R | R | R | R | I | S | S | S | S |
| 101 | M | 30 | ICU |  | A | 348 | R | R | R | R | R | R | R | R | R | I | R | S | I | S |
| 102 | M | 45 | ICU |  | A | 348 | R | R | R | R | R | R | R | R | R | I | S | S | S | S |
| 103 | M | 54 | ICU | Exitus | A | 348 | R | R | R | R | R | R | R | R | R | R | S | S | S | S |
| 75 | F | 41 | ICU |  | B | 348 | R | R | R | R | R | R | R | R | R | R | S | S | S | S |
| 100 | M | 48 | ICU |  | B | 348 | R | R | R | R | R | R | R | R | R | R | S | S | R | S |
| 78 | M | 8 | P-ICU |  | C | 348 | R | R | R | R | R | R | R | R | R | R | S | S | I | S |
| 83 | F | 23 | ICU |  | D | 4844 | R | R | R | R | R | R | R | R | R | I | S | R | I | S |
| 96 | M | 51 | ICU |  | D | 4844 | R | R | R | R | R | R | R | R | R | I | S | S | S | S |

Gdr: gender; PT: pulsotype; MLST: multi-locus sequence type; IPM: imipenem; MEM: meropenem; CTX: cefotaxime; CAZ: ceftazidime; TZP: piperacillin-tazobactam; AMC: amoxicillin-clavulanic acid; ATM: aztreonam; SXT: trimethoprim-sulfamethoxazole; GEN: gentamicin; CIP: ciprofloxacin; CST: colistin; TGC: tigecycline; LVX: levofloxacin; AMK: amikacin; M: male; F: female; ICU: intensive care unit; P-ICU: pediatric ICU. R: resistant; I: intermediate; S: susceptible. ^a^Antimicrobial susceptibility testing was performed by disc diffusion for all antibiotics tested but colistin, that was assessed by broth microdilution. Inhibition diameters were interpreted according to CLSI clinical breakpoints and expert rules for *Enterobacterales* (M100-S29), except for tigecycline, which was interpreted using the European Committee on Antimicrobial Susceptibility Testing (EUCAST) breakpoints and rules for *Enterobacterales* (Version 10.0, January 2020).
