# Supplementary figures and images for: Spread of ST348 Klebsiella pneumoniae Producing NDM-1 in a Peruvian Hospital
Source: Microorganisms. 2020 Sep 11;8(9):1392. doi: 10.3390/microorganisms8091392 (PMC7563475; doi:10.3390/microorganisms8091392)

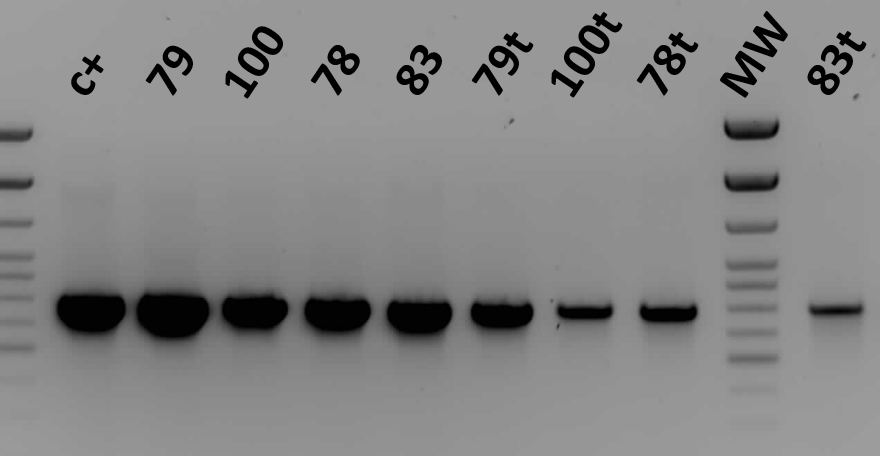

Supplement: Supplementary file 1 [file microorganisms-08-01392-s001.zip › FigS1.tif]
